# Why should multiple dehiscences of the otic capsule be considered before surgically treating patients with superior semicircular canal dehiscence? A radiological monocentric review and a case series

**DOI:** 10.3389/fneur.2023.1209567

**Published:** 2023-08-08

**Authors:** Eugen C. Ionescu, Pierre Reynard, Maxime Damien, Aicha Ltaief-Boudrigua, Ruben Hermann, Gerard J. Gianoli, Hung Thai-Van

**Affiliations:** ^1^Department of Audiology and Otoneurological Explorations, Hospices Civils de Lyon, Lyon, France; ^2^Hearing Institute, Research Center of Pasteur Institute, Team Clinical and Translational Exploration of Sensorineural Hearing Loss, Inserm, Paris, France; ^3^Department of Physiology, Claude Bernard University, Lyon, France; ^4^Department of Radiology, Hospices Civils de Lyon, Lyon, France; ^5^Department of ENT, Cervico-Facial Surgery and Audiophonology, Hospices Civils de Lyon, Lyon, France; ^6^Lyon Neuroscience Research Center, IMPACT Team, INSERM, Centre National de la Recherche Scientifique (CNRS), Lyon, France; ^7^The Ear and Balance Institute, Covington, LA, United States

**Keywords:** otic capsule dehiscence, otic capsule dehiscence syndrome, multiple otic capsule dehiscence, third window syndrome clinical-radiological correlations, third mobile window lesions diagnosis

## Abstract

This review aims to draw attention to the multiple ipsilateral otic capsule dehiscences (OCDs), which may cause therapeutic failure in operated patients. A series of six severely disabled patients with symptoms and signs consistent with a superior semicircular canal dehiscence (SSCD) diagnosis, confirmed by a high-resolution CT scan, is presented here. Five of the patients underwent surgery, and in four of the cases, the postoperative results were poor and/or disappointing. The ethical principles underlying modern medicine encourage medical staff to learn from past experience even when the results are modest despite the accuracy of the treatment applied to a patient. Consequently, we reviewed the radiological records of symptomatic and asymptomatic patients diagnosed or referred to our center for confirmation over the past 5 years to determine the incidence of multiple OCD in this population. Multiple localizations of suspected OCD in the ipsilateral ear did not appear to be rare and were found in 29 of 157 patients (18.47%) in our retrospective review using high-resolution thin-sliced CT scans. The decision to perform surgery for a documented symptomatic superior SSCD should be made with caution only after ruling out concomitant lesser-known variants of OCD in the ipsilateral ear.

## Background

After the first description of superior semicircular canal dehiscence (SSCD) by Minor et al. (1), the understanding of this pathology generated by a third mobile window (TMW) has progressively evolved over time, while the number of reported anatomical variants has increased. Given the similarity of the auditory and/or vestibular signs specifically described in most of these variants, many authors have adopted the concept of a “spectrum of third window abnormalities” (TMWA) (2). This condition is characterized by the fact that it can mimic a considerable number of inner and/or middle ear disorders (3, 4). The recently introduced term “third mobile window syndrome” (TMWS) (5) refers to all pathologies of the TMWA spectrum whose symptoms, clinical signs, and vestibular and audiometric outcomes correspond to bony defects or otic capsule dehiscence (OCD) confirmed or not by high-resolution computed tomography (HRCT) performed according to the recommendations of the Barany Society (5–8).

The above-mentioned approach only suggests the existence of various low-impedance areas of the otic capsule but does not indicate its position or the anatomical elements involved at the dehiscence interface. Therefore, there was recently proposed a three-type anatomical–radiological classification of TMWA (Supplementary material IA), including all known OCD variants (6, 7) (Supplementary material IB). This new classification has the advantage of depicting the anatomical structures that are involved at the level of the abnormal mobile window, which can guide therapeutic management, as has been the case in some labyrinthine-vascular variants (9–12). It also indicates all the existing types of TMWA, including OCDs, multiple OCDs, and CT- or intralabyrinthine, to both ENT and radiology specialists in such a way that this pathology does not remain underdiagnosed. In fact, other OCD variants than SSCD are far from being systematically searched for in the current radiological practice, despite the obvious presence of a TMWS. This can be easily explained because most of the articles communicated or published in the past 20 years have almost exclusively focused on SSCD, although the incidence of these OCD variants is not necessarily lower (13).

Although multiple ipsilateral OCDs have been reported by several authors as case reports (14–21), information on this topic in the literature is still lacking. Only one article addresses this topic in more detail and is not just a case report (22). This article indicates that the frequency of low bone strength area or OCD associations is higher than previously thought. It reports some associations, especially between SSCD and other “classic” dehiscences (such as tegmen tympani or posterior SSCD, geniculate ganglion dehiscence, or variants involving the internal auditory canal and/or posterior semicircular canal (PSC) and the glenoid cavity of the mandible). However, lesser-known and recently described variants of OCD were not cited in this study, such as the dehiscence between the cochlea and the first facial nerve segment (cochlear-facial dehiscence [CFD]), between the lateral semicircular canal (LSC) and the tympanic segment of the facial nerve (LSC/FN) (23), or the vasculo-labyrinthine OCDs involving the internal jugular vein (IJV), the vestibular aqueduct (VA), the cochlear aqueduct (CA), or other previously reported variants (7, 8). We also reported in a recent article, a series in which 11 of 97 patients (11.3%) presented with symptoms confirming multisite OCDs (6). From our analysis of these patients, the most frequent associations appear to be between Type I and Type III OCD variants (such as SSCD and cochleo-facial or LSC/FN), followed by Type II and Type III (such as IJV/VA and CFD or LSC/FN). Previously, Wackym et al. reported a comparative percentage of double ipsilateral locations in a larger radiological study (9.18%) (24). Interestingly, the prevalence of the most frequent associations with OCD variants appears similar in both studies cited above.

We previously stated that the main challenge of multiple ipsilateral OCDs seems to be designating an appropriate treatment strategy in patients with disabling symptoms (6). This includes establishing the order in which these dehiscences should be treated and whether this should be done sequentially or simultaneously. To the best of our knowledge, no current data in the literature guide practitioners to a suitable therapeutic approach regarding multiple OCD. Therefore, the main purpose of this article is to emphasize the fact that the presence of a “classic” and large SSCD on HRCT might dissuade radiologists and neurotologists from searching for further OCDs (i.e., a “distracting lesion”), especially when the symptoms are consistent with a TMWA. Multiple OCDs might remain undiagnosed, which could lead to poor outcomes.

## Methods

The investigation adhered to the principles of the World Medical Association Declaration of Helsinki.

### Radiologic study

Between January 2019 and December 2022, 1,283 patients underwent HRCT of the temporal bone at our tertiary referral center. In most cases, the patients were recommended for HRCT for symptoms such as conductive or sensorineural hearing loss, including profound hearing loss, with or without vertigo or dizziness, that were found during an initial ENT consultation. HRCT records of adult candidates for cochlear implantation were therefore also considered. The files were reviewed by a neurotologist and a radiologist specializing in neurotology, and the cases of suspected multiple ipsilateral localization were unanimously selected as such.

Two hundred forty-five radiologically confirmed or suspected SSCD patients were identified using targeted medical statistical research software. Patients who have undergone middle ear surgery or had chronic inflammatory, neoplastic, or degenerative inner and/or middle ear pathologies were excluded. After applying the criteria, the radiological records of 157 patients (64 men and 93 women) and 314 ears were finally included in this study.

A high-resolution CT scan (GE GSI Revolution, GE Healthcare, USA) of the temporal bone was performed in all patients. As recommended, the slices were acquired helically in the axial plane at a nominal thickness of 0.625 mm with a 50% overlap of 0.312 mm in a 60-mm field of view with a 512 matrix for an isometric voxel [1]. Images were obtained in ultrahigh resolution at 140 kV and 200 mAs/section. Primary images were reworked in the axial and coronal planes of the SSC, LSC, or PSC. Each plane was performed with a 0.2 mm thickness and a 0.2 mm increment using Advantage Workstation Server visualization software (GE Healthcare, USA). Thus, the Pöschl plane was also used to better identify SSCD variants (i.e., the superior plane of the SSC). Other thin multiplanar reconstructions (0.2 mm thickness and 0.2 mm increment) were performed to better identify OCD variants on orthogonal structures of the otic capsule suspected dehiscent. At least two orthogonal thin reconstructions were used in each case of peri-petrous OCD variants (Type III), which usually, in our experience, are not searched systematically as the other more common types of OCDs with the usual HRCT protocol (axial and coronal planes of the second turn of the cochlea of the vestibule, VA, CA). These specific planes were essential for identifying most peri-petrous Type III OCD variants (e.g., CFD and LSC/FN). Inverting the contrast in these cases could provide better recognition of these variants, even if the distinction between “real” and “near” dehiscences can sometimes be challenging (24).

### Case series

The main clinical, audio-vestibular, and radiological elements are presented in this series of six patients with multiple OCD localizations. Although the Dizziness Handicap Index (DHI) and Tinnitus Handicap Inventory (THI) questionnaires were completed before and after the surgical intervention in some cases, these evaluations were not systematic.

### Audio-vestibular assessment

Initial and postoperative audio-vestibular assessments were routinely performed in all patients in the case series reported here and in patients included in the Review section. The audio-vestibular postoperative evaluation was initially scheduled for 3 months, but in cases of symptom resurgence, such as intense vertigo and/or postoperative hearing loss, the patients were examined in a much shorter time interval, from 1 to 2 days up to 2 weeks after the surgery.

The protocol included clinical examinations as well as auditory and vestibular evaluation as follows: pure tone audiometry (PTA; Madsen Astera-Otometrics), middle ear reflexes (Madsen Zodiac 901 tympanometer), videonystagmography (VNG, Ulmer System^®^ Synapsis SA), video head impulse test (VHIT, ICS Impulse^®^ GN Otometrics), and cervical and ocular vestibular evoked myogenic potentials (VEMP, only in selected patients; Bio-Logic^®^ Nav-Pro system) in air conduction (AC) stimuli with 750 Hz tone bursts.

### TMWS simplified score

To identify whether a correlation existed between the level of functional impairment or symptoms and the number of documented uni- or bilateral cases of OCD in each subject, we used a simplified audio-vestibular score. This score was obtained from the medical records of the patients included in the retrospective radiological study. It assesses the most significant signs and symptoms of a TMWA as follows:

Auditory symptoms (1 point each): autophony; pulsatile tinnitus; conductive hearing loss. Vestibular symptoms (1 point each): the presence of Tullio phenomenon or pressure hypersensitivity in the external auditory canal (EAC); physically exerted vertigo or by Valsalva maneuver; permanent or episodic dizziness.

The maximum score obtained was 6, corresponding to a severely impaired patient, and the minimum score, corresponding to an asymptomatic patient, was 0.

## Results

### Radiologic and clinical retrospective review

**Figure 1:**
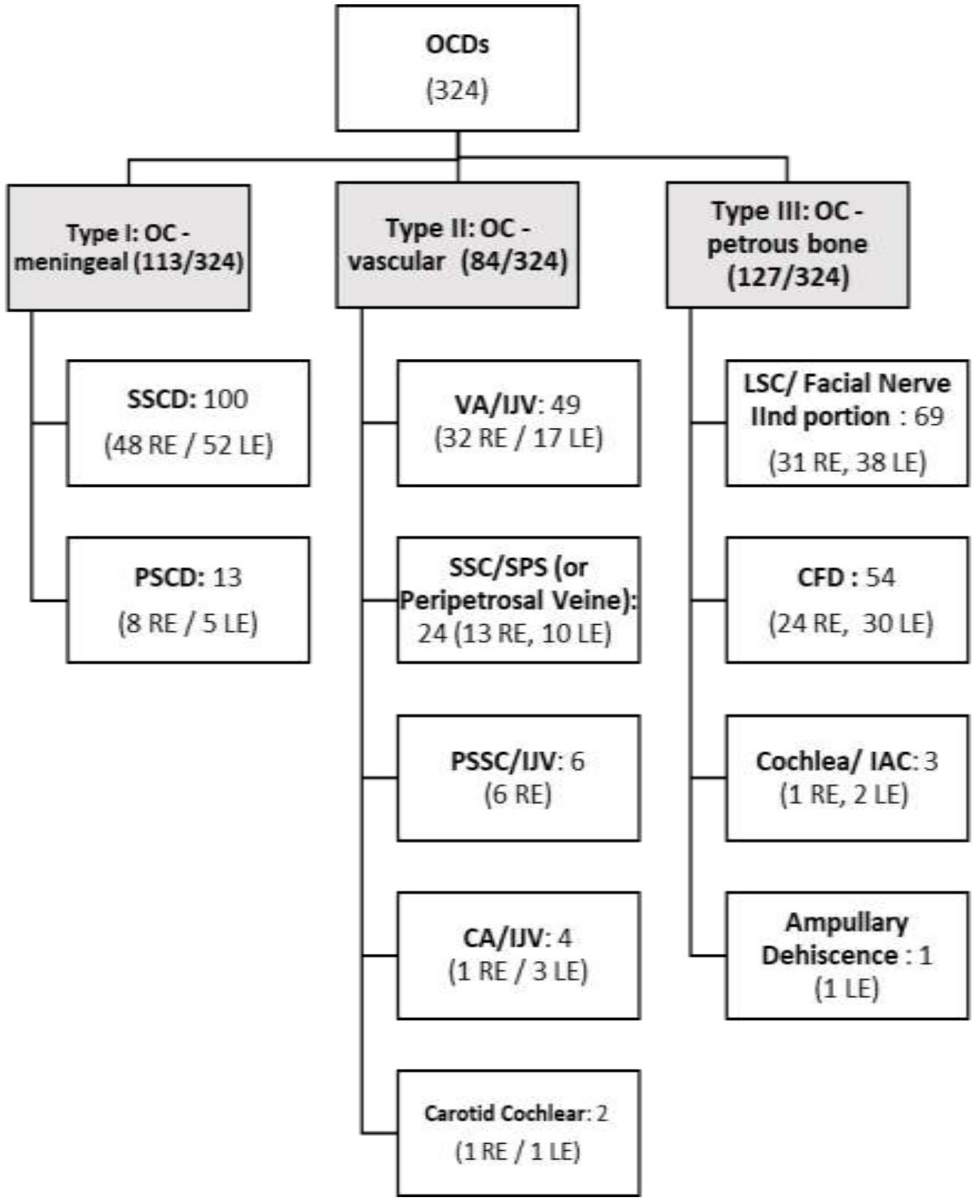
Distribution of OCD by type according to precedent classification in 324 OCDs (157 patients or 314 ears).

Imaging records of 157 patients (64 men and 93 women), or 314 ears, were included in this study. The youngest subject at the time of the HRCT scan was a 6-year-old girl, while the oldest was an 88-year-old woman. Out of 157 patient files, we found a total of 324 OCDs. The distribution by type of OCD (6, 7) in number and affected ear was as follows (Figure 1):

**Type I** (*labyrinthine–meningeal*) accounted for a total of 113 dehiscences out of 324 (34.87%). Of these, 100 were SSCD [48 right ear (RE), 52 left ear (LE)], and 13 were posterior semicircular canal dehiscence (PSCD) (8 RE, 5 LE).
**Type II** (*labyrinthine–vascular*) accounted for 84 dehiscences out of a total of 324 (25.92%): 49 of them were variants localized between the VA and the IJV (32 RE, 17 LE); 23 variants localized between SSC/superior petrosal sinus (13 RE, 9 LE); and 1 less common variant, SSC/subarcuate vein or artery crossing SSC, was found in 1 patient (LE); 6 variants were localized between PSC/IJV (all on the RE); 4 CA/IJV (1 on RE, 3 on the LE); and 2 cochlear-carotid dehiscence variants (1 on each side in the same patient).
**Type III** (*labyrinthine-peripetrosal structures*) accounted for 127 dehiscences out of 324 (39.19%). LSC/FN variant was the most frequent: 69 cases (31 RE, 38 LE); 54 were CFD variants (24 on the RE, 30 on the LE); 3 were cochlea–internal auditory canal dehiscence variants (1 RE, 2 LE); and 1 ampullary dehiscence (LE).

Unilateral and single localizations of OCD were present in 55 out of 157 patients. Twenty patients were classified as Type I OCD (10RE, 10LE). Twenty-six were classified as Type II OCD, including 12 SSC/SPS OCDs (6RE, 6LE) and 14 VA/IJV OCDs (9RE, 4LE). Nine patients were classified as Type III OCD, including 7 patients with CFD (2RE and 5LE) and 2 patients with OCDS involving the LSC and the tympanic segment of the FN (1 RE and 1 LE).

**Figure 2:**
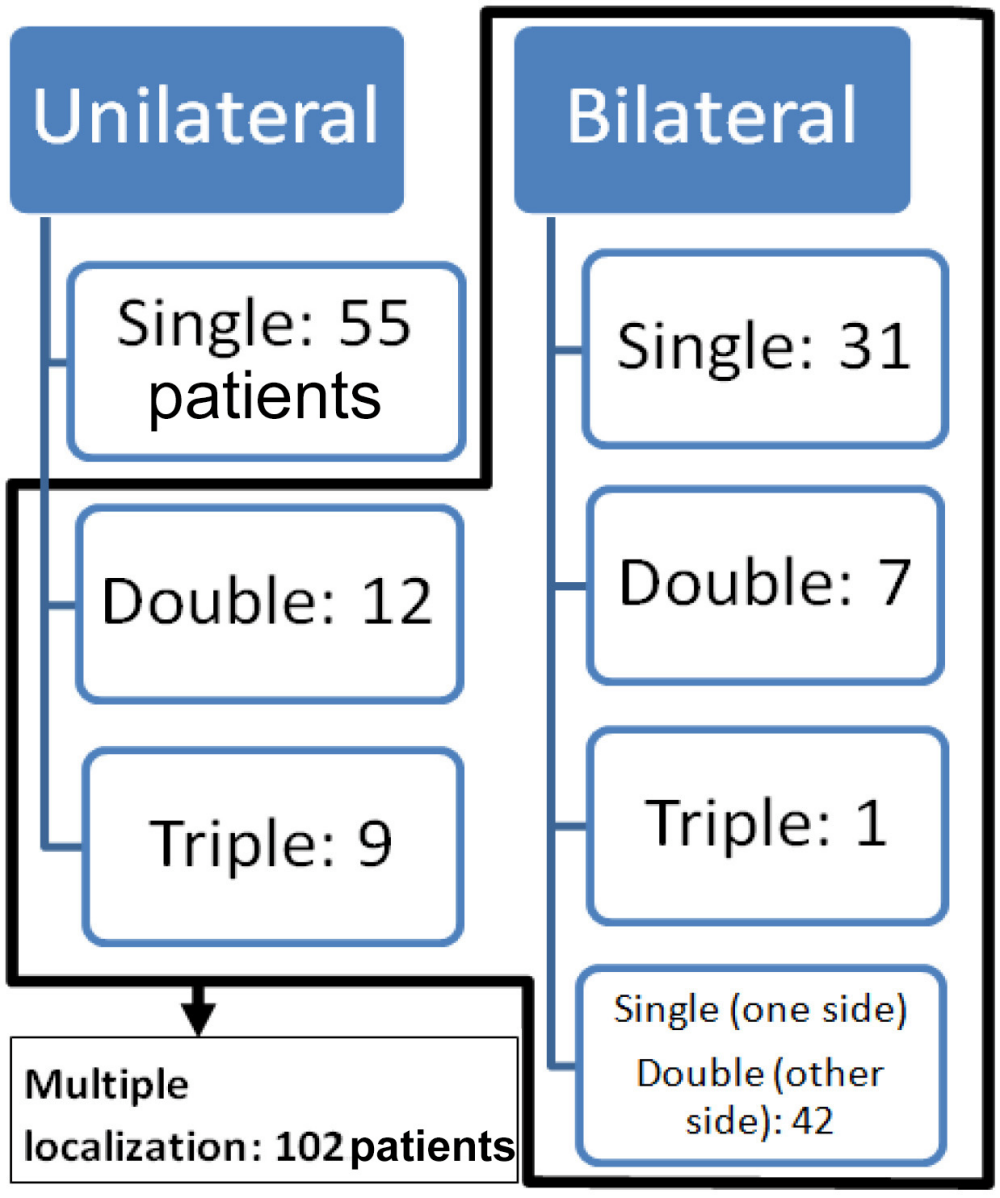
Distribution of OCD between uni- or bilateral and single or multiple OCD localizations in all 157 patients.

Multiple localizations (uni- and/or bilateral) were found in 102 patients (Figure 2). There were 12 patients with unilateral double OCD and 9 with unilateral triple OCD localization. In 39 patients, we found a bilateral localization: 31 patients had a bilateral single localization, 7 patients had a bilateral double OCD, and only 1 had a bilateral triple OCD (Additional Material II). In the remaining 42 patients, we found 126 OCDs arranged in different combinations bilaterally.

### Symptoms and score

Using data from clinical observations, as those patients did not complete the THI or DHI questionnaire, we retrospectively assessed the level of perceived functional hearing and vestibular impairment with a simplified assessment tool. The mean TMWS score obtained in the three groups was as follows (Table 1):

(a) TMWS score in the strictly *single* (*unilateral*) OCD localization group: 2.8 (55 patients),
(b) TMWS score in the *multiple unilateral* localization group: 3.4 (21 patients),
(c) TMWS score in the *bilateral* localization (single and/or multiple) group: 3.98 (81 patients).

**Table 1:** Clinical mean score for each OCD subgroup.

	**Single, unilateral OCD**	**Multiple, unilateral OCD**	**Bilateral (single or multiple) OCD**
Number of patients	55	21	81
Clinical score for TMWS	2.8	3.4	3.98

### Case report series

A table with the essential data of these case reports can be found in Supplementary material III.

#### Case report 1

**Figure 3:**
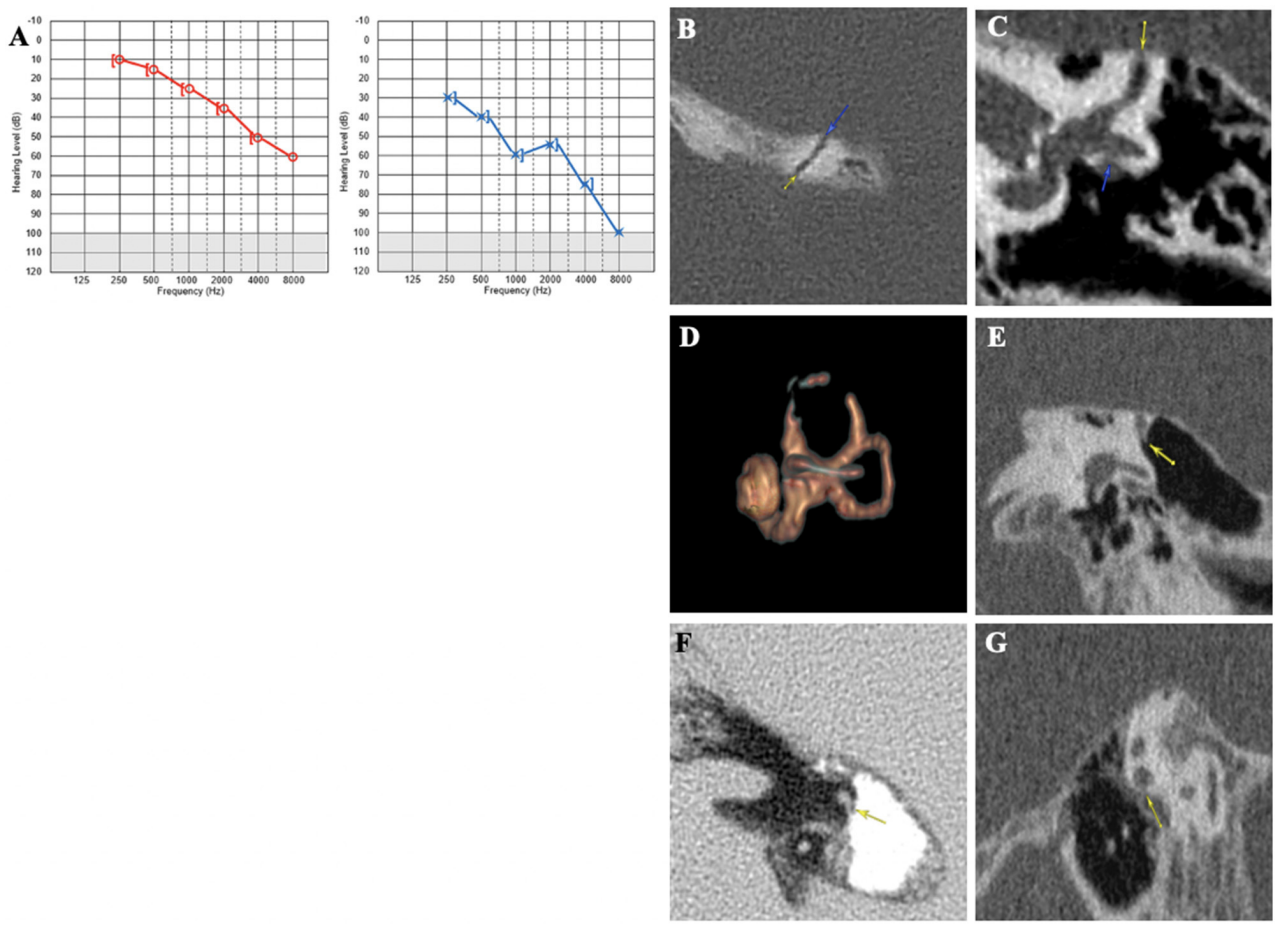
**(A)** Tonal audiometry showing a bilateral sensorineural hearing loss, more pronounced on the left side. **(B, C)** Left SSCD in axial **(B)** and coronal plane **(C)** and CFD (blue arrow) **(C)**. **(D)** Post-operative 3D MRI showing a correctly plugged superior canal. **(E–G)** Post-operative HRCT control showing CFD and another location of SCCD near the surgical approach (yellow arrow).

A 75-year-old patient was examined for hyperacusis in the left ear (LE) and sudden-onset intermittent pulsatile tinnitus. He also described rotatory vertigo triggered by loud noises. The physical examination, including otoscopy, was normal. The audiometric assessment confirmed a pure bilateral sensorineural hearing loss, more pronounced on the LE (Figure 3A). cVEMPs showed a greater response on the LE, where the thresholds were at 80 dB nHL. Caloric tests showed significant left vestibular hypofunction. VHIT gain was normal for LSC but weaker for both left vertical semicircular canals. The HRCT confirmed the presence of a left SSCD (Type I OCD; Figures 3B, C). Because of the disabling and recurrent nature of his vestibular complaints, a left SSCD plugging was performed by transmastoid approach. Unfortunately, the hearing thresholds on the operated side increased to 80 dB nHL on all frequencies after the operation and did not improve. 10 months after surgery, after a period of improvement, the patient described a recurrence of rotatory vertigo and pulsatile tinnitus. Postoperative 3D magnetic resonance imaging (MRI) confirmed the expected absence of an endolymphatic signal, proving that the left SSC was rightly plugged (Figure 3D). When reviewing the postoperative HRCT control, two Type III OCDs could be observed: one involving the LSC and tympanic segment of the FN (LSC/FN) (Figures 3C, G), unknown in the literature at the time of the surgery, and a second dehiscence near the operated area probably due to the drilling during mastoidectomy (Figures 3D, F). The patient was lost to follow-up.

#### Case report 2

**Figure 4:**
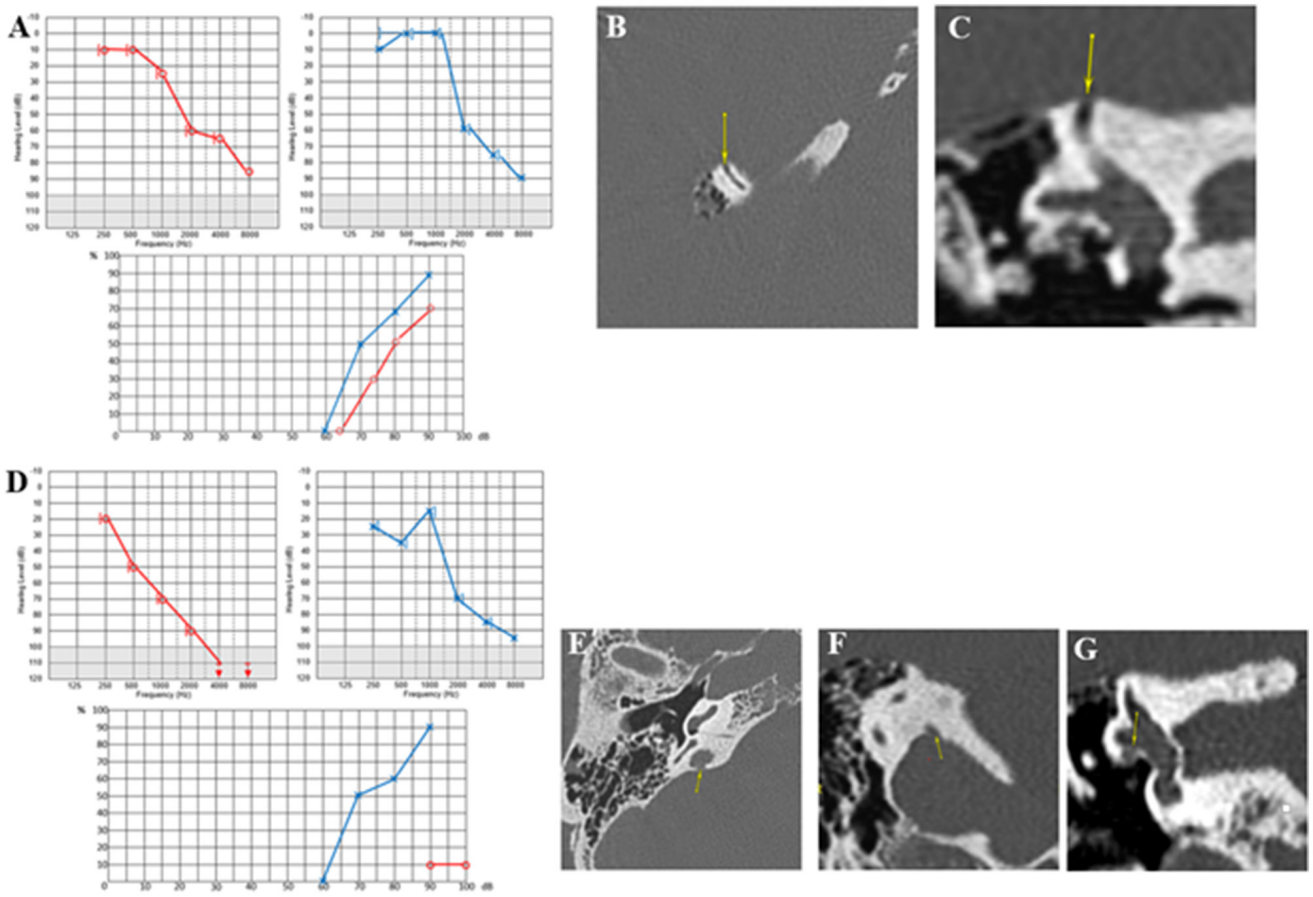
**(A)** Tonal and vocal audiometry showing a bilateral sensorineural hearing loss. **(B, C)** Right SSCD in axial **(B)** and coronal plane **(C)**. **(D)** Post-operative audiometry showing a major deterioration of intelligibility. **(E, F)** Right OCD involving vestibular aqueduct and intern jugular vein in axial **(E)** and coronal plane **(F)**. **(G)** Right OCD between LSC and FN (yellow arrows in **B, C, E–G** indicating various OCD).

A 74-year-old patient presented with autophony in the RE associated with a Tullio phenomenon and effort-induced dizziness with closed glottis. The patient also complained of perceiving sounds coming from the cervical spine in the RE through neck movements. The PTA showed a bilateral sensorineural hearing loss, more pronounced on the RE, with no conductive component and very poor intelligibility (Figure 4A). cVEMPs showed very large responses, with a detection threshold of 50 dB nHL on the RE. VNG showed a slightly decreased vestibulo-ocular reflex (VOR) gain on kinetic tests and a right hypofunction on caloric testing. HRCT confirmed a right SSCD (Type I OCD; Figures 4B, C). The patient underwent an SSCD occlusion through a middle fossa approach. Within a month, he described a significant recurrence of symptoms, while audiometry showed a decrease of 30 dB in low and mid frequencies. In the third postoperative month, this PTA threshold's degradation on the operated side was doubled by major deterioration of intelligibility and autophony reappearance (Figure 4D). This subject was also lost to follow-up.

However, the HRCT image review noted two additional unidentified OCD localizations ipsilaterally: (1) a Type II labyrinthine-vascular dehiscence between the VA and the IJV (yellow arrow) (Figures 4E, F) and (2) a Type III OCD between the Lateral semicircular canal (LSC) and the tympanic segment of the Facial Nerve (FN) (Figure 4G).

#### Case report 3

**Figure 5:**
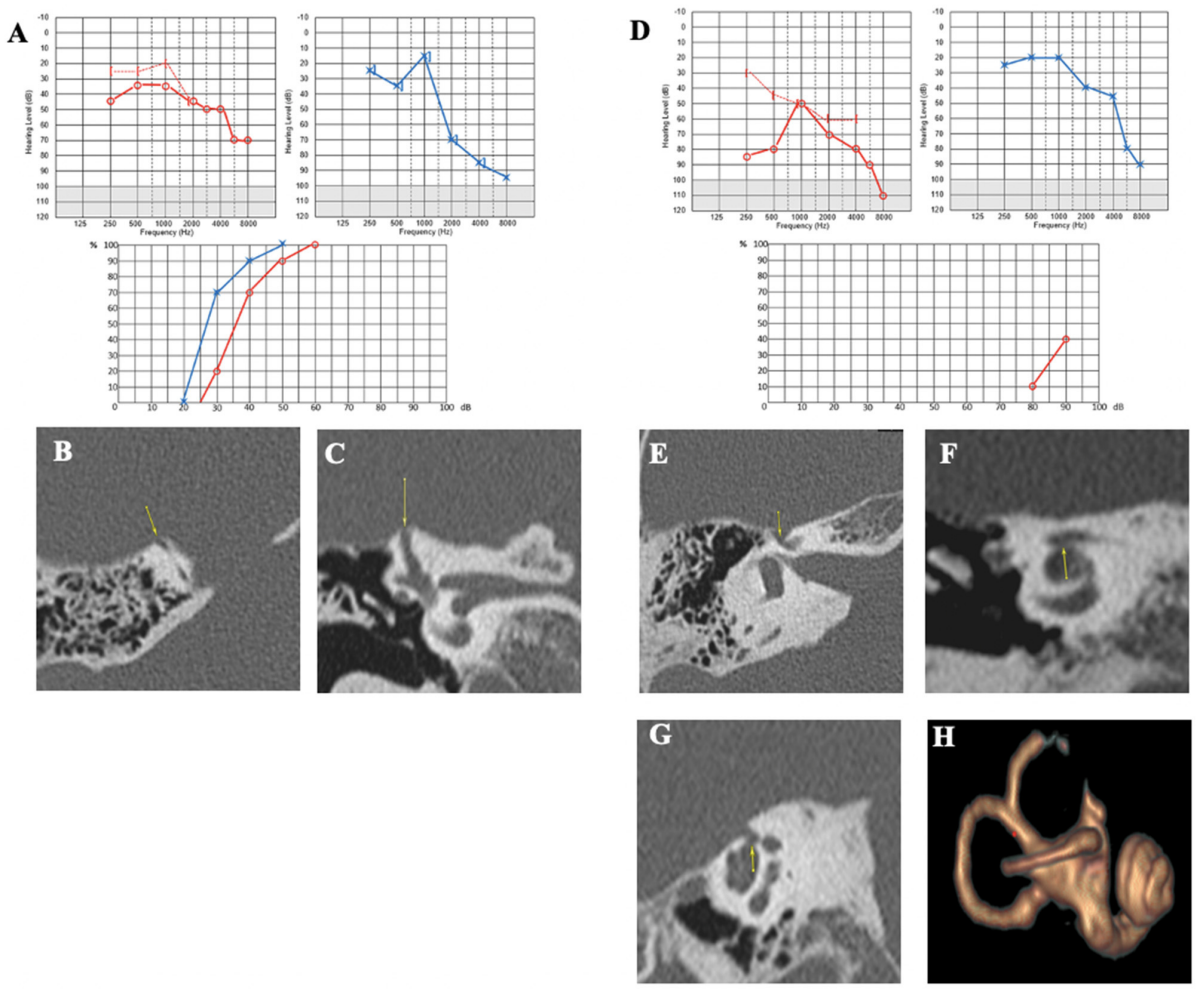
**(A)** Tonal and vocal audiometry showing a bilateral sensorineural hearing loss, with conductive component on the right side. **(B, C)** Right SSCD in axial **(B)** and control plane **(C)**. **(D)** Post-operative audiometry. **(E, F)** Right CFD in axial **(E)**, coronal **(F)** and sagittal plane **(G)**. **(H)** Post-operative 3D MRI showing a complete obliteration of SSCC.

A 70-year-old patient presented with persistent dizziness associated with hearing loss, intermittent autophony, and pulsatile tinnitus on the RE. He also complained of perceiving cervical and temporomandibular joint movements and blinking in the RE. The symptoms, which included dizziness and intense vertigo, began with a lung infection associated with coughing and intense sneezing. The vestibular symptoms seemed to be in sync with the acts of coughing and nose blowing. However, exposure to loud noises did not seem to generate the Tullio phenomenon. PTA showed mild presbycusis with a moderate conductive component at 0.25 and 0.50 kHz on the RE, with preserved bilateral intelligibility (Figure 5A). The cVEMPs showed a significant decrease in the detection threshold up to 70 dB nHL on the RE; the oVEMPs were remarkably large on the RE and absent on the LE. The caloric tests showed a slight left vestibular impairment. The HRCT scan confirmed the presence of a right SSCD (Figures 5B, C). Due to the severity of vertigo (DHI quoted 62/100), a right SSCD occlusion was performed using the transmastoid approach. On day 2, the patient complained of hearing loss and a stronger and more persistent resonance on the operated side than previously. Treatment with oral corticosteroids was prescribed for 10 days to protect and improve the cochlear function. Audiometry showed hearing loss on all frequencies in the RE, with a more pronounced low-frequency air-bone gap than before the surgery (Figure 5D). The vestibular assessment showed a right irritative horizontal nystagmus, inhibited by ocular fixation and unresponsive to applying 100 Hz bony vibrations. VHIT showed a normal gain for all semicircular canals. 3 weeks later, dizziness decreased during physical exertion, nose blowing, or coughing. The irritative nystagmus described earlier had disappeared. However, right hearing loss and autophony persisted. Although the PTA slightly improved compared to day 2, pulsatile tinnitus increased, and oVEMPs completely disappeared. The DHI score showed significant improvement with a score of 28/100 (compared to 60/100 before the surgery), but the THI score showed worsening of the tinnitus with a score of 50/100 (compared to 28/100 before the surgery). HRCT image reexamination noted a CFD I that was undiagnosed before surgery (Type III) (Figures 5E–G). However, MRI confirmed a good obliteration of the right SSC (Figure 5H).

At 6 months, the patient no longer had incapacitating vertigo but continued to experience very brief, non-positional vertigo intermittently. Aural fullness and autophony were less intense but still present. Audiometry showed a slight decrease in the hearing threshold with preserved intelligibility. The vestibular assessment showed no response to cVEMPs on the RE and a decreased VOR gain in the right SSC at VHIT.

#### Case report 4

**Figure 6:**
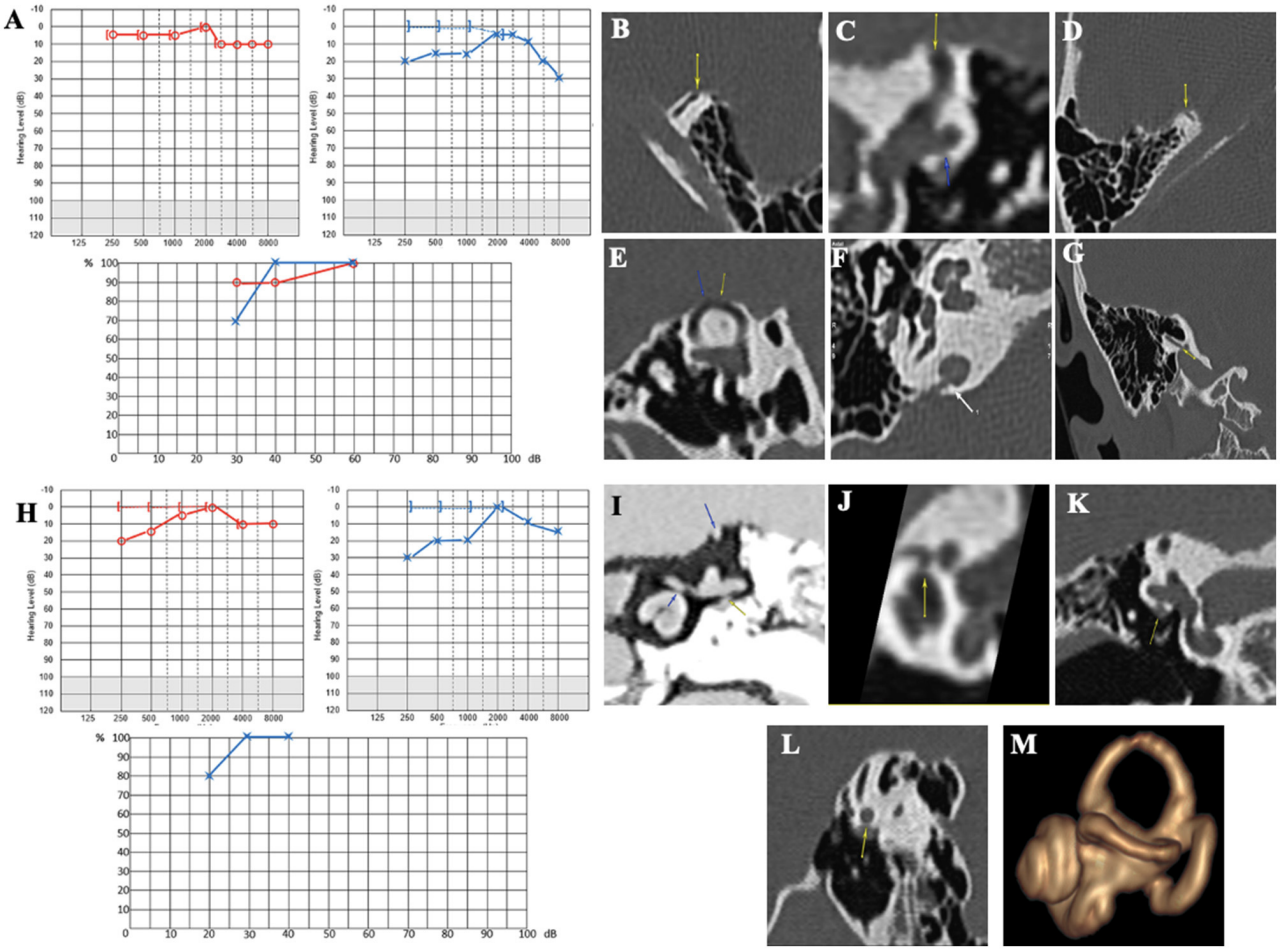
**(A)** Tonal and vocal audiometry showing a mild conductive hearing loss on the left side. **(B, C)** Left SDC in axial **(B)** and coronal plane **(C)**. **(D, E)** Right SDC in axial **(D)** Pöschl plane **(E)**. **(F, G)** Dehiscence between right IVJ and vestibular aqueduct in axial **(F)** and coronal plane **(G)**. **(H)** Post-operative tonal and vocal audiometry. **(I)** Coronal plane in negative contrast showing near-cochleofacial dehiscence, near-dehiscence between LSC and FN II and SSCD. **(J)** Near-cochleofacial dehiscence. **(K, L)** Near-cochleofacial between the right LSC and FN II in coronal **(K)** and sagittal plane **(L)**. **(M)** Post-operative 3D MRI showing an effective capping of SCC.

A 32-year-old patient was referred to our department by a fellow neurologist for left pulsatile tinnitus and positional vertigo. An MRI raised the suspicion of cross-compression syndrome of the VIII cranial nerve by a vascular loop. The patient, presenting with a migraine history, also complained of persistent spontaneous dizziness or short vertigo from physical exertion. She was prescribed an anti-migraine treatment with non-steroid anti-inflammatory drugs, which, according to her, were only taken for intense headache crises. She also reported eye movement perception in the LE. Audiometry confirmed a left low-frequency conductive hearing loss (Figure 6A). Auditory brainstem responses were found to be normal. Caloric and rotatory tests were normal as well. cVEMPs showed very large responses, with a detection threshold identified at 60 dB nHL on the LE. The HRCT confirmed a left SSCD (Figures 6B–D). On the RE, a smaller SSCD Type I was identified (Figure 6E) associated with Type II OCD (VA/IJV) (Figures 6F, G); on this side, the patient only complained of intermittent tinnitus. Given the high THI score (over 70/100), a resurfacing surgical technique of the left SSC using a transmastoid approach was decided. On day 1, an intense left horizontal nystagmus was observed. Audiometry showed a lateralized Weber test to the LE with no increased BC threshold. Postoperative HRCT on day 2 revealed air bubbles at the top of the left SSC. Dizziness gradually subsided under symptomatic treatment. For ~3 months, the patient complained of a decrease in intelligibility and fullness in the operated ear. At 4 months, she began to complain of noise reappearance in the operated ear, along with a disturbing perception of eyeball movements. Pulsatile tinnitus and vertigo sensitive to stress, fatigue, or loud noises reappeared. Audiometry revealed a deterioration of the thresholds in AC in both ears (Figure 6H). A postoperative vestibular assessment revealed normalization of the cVEMPs, thereby presenting in favor of a technically successful surgery. However, the DHI score was rated at 76/100 and the THI at 82/100, both higher scores than before the surgery. Reexamination of the HRCT showed an additional near-dehiscence Type III (between the right LSC and the tympanic segment of the FN in the LE) (Figures 6I, J). A similar localization was also identified on the RE (Figures 6K, L). The MRI showed a well-performed surgical capping technique without any complications (Figure 6M). Once again, the patient was lost to follow-up.

#### Case report 5

**Figure 7:**
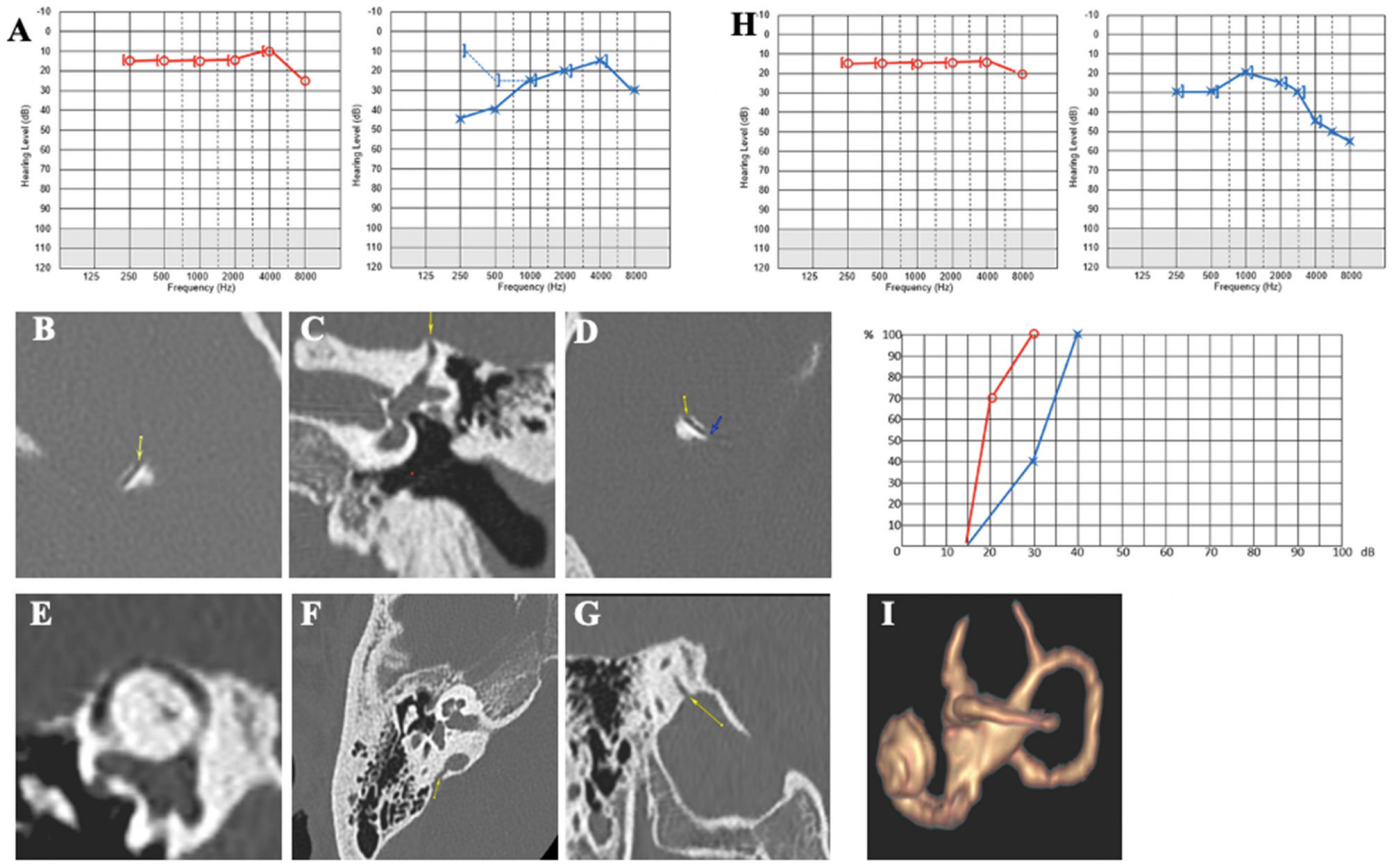
**(A)** Tonal and vocal audiometry showing a mild conductive hearing loss on the left side. **(B, C)** left SDC in axial **(B)** and coronal plane **(C)**. **(D, E)** Right SDC in axial **(D)** Pöschl plane **(E)**. **(F, G)** Dehiscence between right IVJ and vestibular aqueduct in axial **(F)** and coronal plane **(G)**. **(H)** Post-operative tonal and vocal audiometry. **(I)** Post-operative 3D MRI showing an effective plugging of SCC.

A 58-year-old patient was referred to our department for autophony and resonance in the LE (heartbeat, footsteps, and eyeball and eyelid movement perception) and left pulsatile tinnitus. Audio-vestibular assessment revealed left conductive hearing loss (Figure 7A) and cVEMP with a very large amplitude and abnormally low bilateral thresholds (70 dB nHL on the RE and 60 dB nHL on the LE). The rest of the vestibular assessment was normal. CT scan showed a bilateral SSCD (Figures 7B–E), with an additional OCD between the VA and the IJV on the right non-symptomatic ear (Figures 7F, G). An SSCD plugging was performed by the middle fossa approach.

At 4 months, the pulsatile tinnitus had disappeared. Conductive hearing loss, autophony, and resonance symptoms in the LE improved. The cVEMPs normalized on the LE. The patient reported improvement in both autophony and pulsatile tinnitus but complained of a new but less severe tinnitus, probably due to high-frequency hearing loss (Figure 7H). MRI showed that the SSCD had been properly plugged (Figure 7I).

#### Case report 6

**Figure 8:**
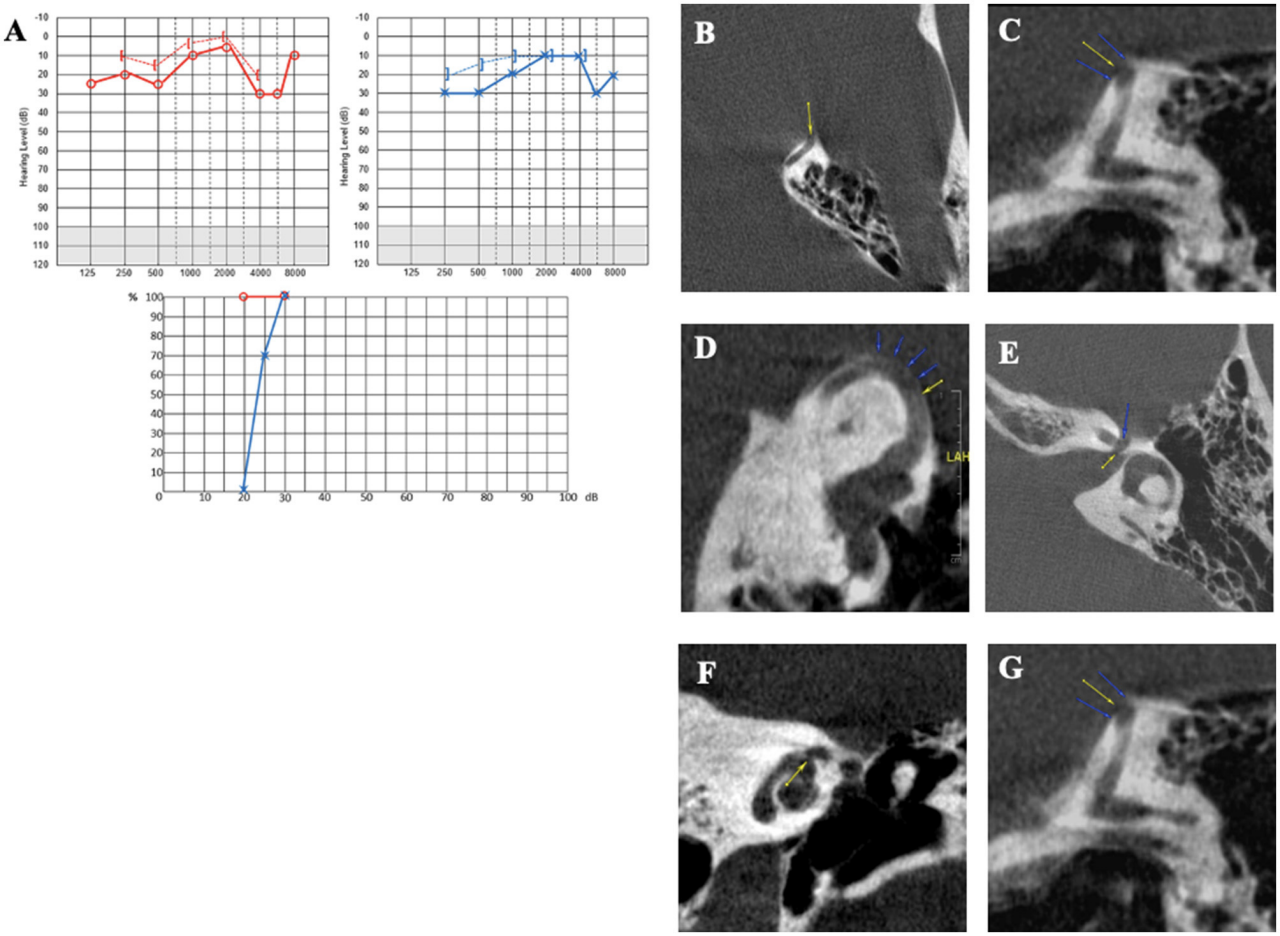
**(A)** Tonal and vocal audiometry showing a slight bilateral conductive hearing loss. **(B–D)** Left SSCD in axial **(B)**, coronal **(C)** and Pöschl plane **(D)**. **(E, F)** Left cochleofacial dehiscence in axial **(E)**, and coronal plane **(F)**. **(G)** Left SSCD in a control plane.

In this 49-year-old patient, symptoms were triggered following acoustic trauma by exposure to a loudspeaker. Despite a 7-day course of steroid therapy, he continued to complain of bilateral pulsatile tinnitus, more pronounced on the LE, where a painful fullness and autophony were felt even with mild or medium intense sounds. There was no vertigo, just a slight but almost permanent dizziness. The clinical examination was normal. Audiometry initially revealed bilateral low- and medium-frequency conductive hearing loss (greater on the left side), with a minimally bilateral 4 kHz notch. A tinnitus assessment could not accurately indicate the characteristics of bilateral tinnitus (Figure 8A). HRCT showed SSCD (Type I OCD) on the LE (Figures 8B–D) and a very thin bony covering on the RE, suggesting a “near” SSCD. THI was rated at 90/100. After 3 months of follow-up, audiometric tests showed that the conductive hearing loss had disappeared on the RE and diminished on the LE. Cervical and ocular VEMPs were consistent with an SSCD on the LE. The patient still did not complain of vestibular symptoms, and the chronic dizziness gradually disappeared. HRCT images showed a second OCD (a CFD variant) (Figures 8E, F) on the LE. We also noticed that the SSCD on the LE was located on the anterior slope (arrows) and not “typically” placed on the convexity of the bony SSC (Figure 8G). Considering the experience from similar Case 3, the patient followed our advice and did not undergo surgery, tinnitus being the dominant symptom in his case. A hearing aid was fitted to the left ear combined with an ipsilateral white noise tinnitus masking. This resulted in progressive improvement in symptoms. After 6 months, the THI fell to 60/100, and the patient no longer used anxiolytics or other medication to fall asleep.

## Discussion

### Retrospective review

After analysis of various medical records, since THI or DHI questionnaires were not available, we retrospectively rated the severity of auditory and/or vestibular symptoms with a simplified assessment tool (Table 1). When combining the higher incidence of multiple localizations and the higher audio-vestibular discomfort score in multiple OCDs, it appears that the higher the score (in a severely impaired patient), the greater the likelihood of finding multiple dehiscences. Further studies targeting patients operated on with a poor or disappointing postoperative result could support these observations.

The reason behind such a difference between the number of diagnosed SSCDs and the total (much larger) number of OCDs observed after systematic radiological reexamination is that many otologists and radiologists seem to mainly focus on the most common variants of otic dehiscence (e.g., SSCD or PSCD) and ignore or be unaware of the existence of other lesser-known variants. Therefore, radiologists trained in otology should be informed and aware of possible multiple locations, but they should also be more familiar with the recently described variants of OCD. Likewise, otologic surgeons should consider the possibility of multiple OCDs before proposing a surgical approach for what initially looks like a typical SSCD. For multiple dehiscences that can be accommodated, such as a combined SSCD and PSCD, concomitant surgical repair may be considered. For many of the lesser-known OCDs, such as CFD and horizontal semicircular canal-FN dehiscence, there is no direct surgical repair of the dehiscence at present. However, the identification of these lesions preoperatively notifies the surgeon of the possible use of window reinforcement during surgery (24).

In a general way, all ENTs should be informed that OCD is not as rare as it was believed a few years ago. The John Hopkins histological study carried out on more than 1,000 temporal bones shows that 1.5% of specimens have extreme bony thinning (25). Furthermore, SSCD and CFD, defined together with “near” variants, accounted for 6.5% of the general population (26). It could be added that many of the SSCD cases are probably not diagnosed because the ENT, in the absence of conductive hearing loss, as one can observe in some TMWA, only obtains an MRI for the patient who complains of autophony and dizziness, but not an HRCT. Thus, the diagnosis can be missed, especially in cases of lesser-known OCD variants. That is to say that a normal audiogram is not sensitive to TMWS, and consequently, it is not a good enough screening test to exclude TMWS. Another possible cause of underdiagnosis or misdiagnosis of OCDs is that many audiologist technicians or even ENT specialists do not insist enough on looking for the real auditory thresholds in bone conduction (BC). This is even more true when the air conduction and the tympanogram with the study of middle ear reflexes are normal. Thus, it is possible to pass along and not detect a supra-normal auditory threshold in bone conduction, which can indicate a TMW lesion.

### Case series

We presented a series of six patients with symptomatic SSCD. Five of them underwent surgery, while in one patient we advised against the originally planned surgery. In four of the five operated patients, the postoperative results were considered unfavorable or disappointing. After reviewing the radiological and clinical files, we can speculate that the treatment failure in these patients may be due not only to surgical technique errors, as can be discussed in Case 1, but also possibly and very likely to the simultaneous presence of other OCDs in the treated ear that had not been diagnosed before surgery.

This is also the reason why the sixth patient, initially scheduled for a plugging operation, did not undergo surgery because the reexamination of the HRCT images revealed the presence of an ipsilateral CFD in addition to the previously diagnosed SSCD.

This finding led us to analyze additional records, thus completing the initial objective of this article. Therefore, all radiological records of patients diagnosed with SSCD in our center during the last 5 years were reviewed. The results, including all types of otic dehiscence known at the time of submission, were reported in the order of their incidence and according to their type, following the classification of Reynard et al. (6).

In patients 1 and 2, no typical conductive hearing loss was found (Figures 2A, 3A), as may be expected in symptomatic SSCD. Patient 1 presented a profound postsurgical neurosensorial hearing loss, despite what would appear to be a well-performed left SSC plugging on the postoperative MRI (Figure 3D). However, fullness and vertigo triggered by loud sounds reappeared in the operated LE. After analysis of the postoperative radiological images, the existence of a second dehiscence (or near dehiscence) between the left LSC and the FN in its second segment (LSC/FN) was suspected (Figure 3C – blue arrow and G – yellow arrow). A third OCD was also observed on the same LE, which was probably iatrogenic and mainly contributed to the symptom's reappearance (Figures 3E, F). Although postoperative pure-tone hearing loss did not occur in patient 2, speech audiometry dramatically decreased after SSCD plugging (Figure 4D). As in Case 1, when the radiological records were reexamined after the surgery, an additional OCD was diagnosed on the operated ear (Figures 4E–G). A possible explanation of the postsurgical hearing loss observed in these two cases could be that the SSCD plugging reduced the volume of the endolymphatic space, generating a hyper-pressure in the membranous cochlear canal and, thus, a significant auditory transduction disruption. This assumption is in accordance with the results of experimental animal models of hydrops (27, 28). Moreover, the apparition of hyper-pressure in the endolymphatic system that seemed to manifest itself immediately after plugging is also supported by the two following cases in which signs of vestibular hydrops were evident.

Patient 3 presented with postoperative irritative nystagmus, significant hearing loss, and other clinical signs suggestive of induced endolymphatic hydrops (EH) persisting for several weeks. An ipsilateral CFD II (Figures 5E–G) was diagnosed after surgery, while a 3D labyrinthine MRI confirmed a well-performed SSCD plugging (Figure 5H).

Patient 4 presented the same clinical signs of secondary hydrops on day 1 after surgery. However, the technique, in this case, was a capping type (Figure 5M), and the Type III additional OCD (LSC/FN) was rather “near” dehiscence (Figures 5K, L). Therefore, it is difficult in this case to attribute the appearance of clinical signs of hydrops to a reduction in the volume of the endolymphatic system that would be minimal with this surgical technique. However, the phenomenon could be partially explained by the local pressure change at the LSC/FN dehiscence level once the impedance at the SSCD interface has been normalized by confinement. On the other hand, the gradual onset of vertigo after surgery could also be due to right-sided OCDs that could have started to manifest themselves clinically. Indeed, the follow-up audiometry showed a right-sided conductive hearing loss, initially absent.

Patient 5 only complained of a transient increase in tinnitus on the LE after surgery. This can also be explained by the appearance of hydrops induced by the SSCD plugging, which was well performed according to the control labyrinthine MRI (Figure 7I). However, in this case, the evolution was good after a few weeks, with a significant improvement in symptoms. In patient 6, who also presented with clinical signs of SSCD, the discovery of additional ipsilateral dehiscence justified the cancellation of the proposed surgical intervention since the main complaints were only tinnitus and ear fullness. In this case, plugging or resurfacing would probably have led, as in Cases 1, 2, 3, and 4, to the exacerbation of symptoms through the expected secondary hydrops. Furthermore, the dehiscence occurred very anteriorly. Therefore, any temporal lobe descent into the membranous SSC would be limited by the bony overhang of the semicircular canal (Figure 8C). This fact, combined with a thicker folded expansion of the dura mater at the interface of the SSC window, would limit its lack of resistance, thus explaining the lack of impairing vestibular symptoms. 3D labyrinthine MRI performed to exclude SSCD autoplugging or other membranous labyrinthine deformations was normal. This patient finally benefited from a medical treatment conducted by a psychologist by combining a tinnitus masker and tinnitus retraining therapy, which has led to the improvement of the patient's symptoms. What also contributed to our recommendations against surgery was that only a very small and transient conductive hearing loss was observed with the PTA, while the cervical and ocular cVEMP thresholds were very low, as in the case of the first two patients in this series. In a recent study, Noij et al. (29) pointed out that the coherence between the bone and air conduction gap at PTA and the cVEMSs threshold is a very useful tool for confirming the diagnosis of symptomatic SSCD. This was verified in patients 1, 2, and 6, in whom the bone gap was initially insignificant (6) or completely absent (as in Cases 1 and 2), as “the indicator of a symptomatic SSCD” was negative. The rest of the audiometric or vestibular examinations (VHIT and VNG) obtained before surgery seem to be of no use to indicate the presence of multiple OCDs in the same ear. These observations suggest that in the absence of the Tullio phenomenon or equivalent vestibular signs or symptoms, together with the absence of conductive hearing loss, but with cVEMPs or oVEMPs indicating a TMWA, the otologist should consider that another variant of OCD on the same ear may be present, apart from an easily noticeable SSCD. On the other hand, the skull vibration test, easy and quick to perform in daily practice under the mask of videonystagmoscopy, can indicate the appearance of a characteristic nystagmus as previously shown (30), thus drawing the attention of the examiner that an OCD may be present. This can be more useful as the rest of the vestibular evaluation through VNG and VHIT returns to normal quite frequently.

Regarding the surgical approach, our team generally preferred the transmastoid route because we consider it more convenient for moderate or small-sized dehiscence. Instead, in Cases 2 and 5, a middle fossa approach (Additional Material III) was used because the SSCD dimensions were larger than in the other three cases and it was considered that a wider approach would ensure better-quality plugging and/or capping.

### A required systematic radiological protocol

In case of clinical and audiological signs or symptoms suggestive of TMWA, the HRCT radiological protocol should first include a careful search for all known variants of OCD (Types 1 and 2). Special attention should be paid to peri-petrous variants of OCD (Type 3) because their size is smaller and, in case of association with a prominent SSCD (or another better-known variant), there is a risk of overlooking or failing to diagnose dual or multiple ipsilateral OCDs. Careful investigation of these variants is also very important, as their incidence appears to be higher than that of other variants (39.07% in this study). These might be harder to find and be considered “symptomatic” because their size is much smaller than the “classic” variants. The grayscale inversion function is especially valuable when small OCD variants (or Type III peri-petrous) are suspected. As for “classic” OCDs, it can be hypothesized that if a special HRCT technique is not applied with slices smaller than 0.6 mm or even 0.5 mm, there is a risk of underdiagnosis of small dehiscences, or, in the opposite sense, overestimating their clinical importance. Presently, in our knowledge of TMW pathology, it is not possible to understand with the currently accepted mechanism models how these small subvariants of OCDs can disturb the cochlear micromechanics, a fact that is frequently observed in these cases (4, 5, 23, 24).

Although there are currently standard criteria in the literature to differentiate a “near” SSCD from a “real” one (31, 32), this is not yet the case for newer and “smaller” variants of OCD. However, we do know that a near SSCD can present clinical and audio-vestibular features of TMWS (32) and that performing surgery in some of these cases could improve them (33). It is also worth noting that positive radiological diagnosis can be negatively impacted by HRCT techniques that often use a 0.6 mm slide, which may be too large for visualizing these smaller variants of OCD. The use of procedures such as cone-beam CT (34) or photon-counting CT, which should improve the contrast-to-noise ratio and spatial resolution (35), would probably be an option in future for better recognition of these variants.

### Therapeutic challenge

Although we currently have guidelines for the diagnosis of SSCD (8), there are no published studies on the criteria for selecting a candidate for surgical intervention (36). Thus, in the case of very symptomatic and disabled patients in everyday life, the expected benefit/risk ratio must be discussed and very well-explained to avoid postoperative disappointments. Although the strength of this study is limited by the analysis of records from a single center and the fact that the number of patients reported is not large enough to support a comparative statistical study, we think that the essential emerging message is still clear. Surgeons should be aware that a cause of surgical failure in a patient with SSCD may be the unnoticed presence of another OCD, even of minimal or “atypical” size. Considering the consistently poor postoperative results in this series of patients with multiple OCDs who underwent technically successful surgeries, it is justified to question whether the currently accepted third window techniques and mechanisms for (single) SSCD (36, 37) also apply to multiple OCDs. It is reasonable to believe the opposite since in the case of several abnormal areas of low impedance at the level of the otic capsule, the dispersion of acoustic energy would be logically greater and more diffuse than in the case of a “single” TMW. This fact could have negative consequences for the normal transmission of acoustic energy, or “traveling waves” (38, 39), to the basilar membrane of the cochlear canal. Because in this case the rigidity of the endolymphatic system is presumably altered, local acoustic phenomena — as for example standing waves or the phenomena of abnormal local resonance (40) — may appear with negative consequences on auditory transduction process. These could therefore generate not only the “classic conductive hearing loss” involving the inner ear but also a potential decrease in intelligibility or auditory distortions, as we have observed in some of our patients with multiple OCDs (Supplementary material II). From a practical point of view, it means that in very symptomatic and disabled patients, the therapeutic decision to perform an SSC plugging should be taken only after the exclusion of another associated OCD. Thus, in the case of ignored multiple OCDs, performing surgery for an SSCD in a patient without clear and specific vestibular symptoms evocative of TMWS (e.g., Tullio phenomenon or pressure sensitivity in the EAC) could worsen the postoperative clinical status of an undiagnosed OCD. This seems obvious because the volume of the endolymphatic system after a plugging procedure is diminished by the surgical procedure, favoring the appearance or persistence of concomitant EH (41). Regarding the EH associated with OCD, it should be added that some authors have reported this entanglement between two apparently distinct pathologies (41–43). Future studies should verify whether the simultaneous presence of a an EH (confirmed by electrocochleography and dedicated imaging), it does not constitute a risk factor for the postoperative results in case of SSCD plugging-type surgery. This appears as obvious, since the physical stress generated by the decrease of the volume of the endolymphatic space after the respective surgical intervention, would logically lead to a additional increase in pressure in the endolymphatic space. In light of clinical symptoms and initial and postoperative vestibular assessments, it is possible that we are talking about just such a subject in the first two case reports of the present series.

To solve all these theoretical and therapeutic dilemmas, future studies should be carried out not only on numerical or animal models but also on physical models of semicircular canals that would allow the simulation of this complex pathology. However, in our opinion, it should be expected in the case of multiple ipsilateral OCDs that the treatment be addressed simultaneously for all OCDs by micro-invasive methods—or at least for those more surgically accessible. An essential role would be the precise identification of the bone defect location as well as its geometry and dimensions. Three-dimensional bone printers would therefore play an important role in future (44). While waiting for a more adapted therapeutic solution, a more aggressive medical treatment, including anti-hydrops drugs, can be tried in very symptomatic patients (avoiding the trigger factors, prescribing diuretics, and/or anti-migraine drugs) (45).

## Limitations of the study

The authors chose to limit the radiological study only to patients with “spontaneous” OCDs. Therefore, patients who underwent previous otological surgery and those suspected of having ear malformations in relation to neoplastic, infectious, or degenerative pathological conditions were excluded. Another limitation is the fact that the total number of surgically treated patients in our center did not allow for a comparison between the postoperative results of the group of patients reported here whose multiple OCDs were undiagnosed before the surgery and the postoperative results of a comparable group of patients who underwent surgery for a certain SSCD diagnosis. The cause is that in our center, the number of SSCD surgeries is still small. Obviously, this should ideally be carried out multicentrically in the future. The purpose of this study is to sensitize fellow neurotologists since multiple OCDs associated with different types of variants are not as rare as one might think. We also must add that the voxels were anisotropic for the HRCT technique, so there may be limitations due to partial volume averaging that could overemphasize the presence of anatomic dehiscence.

## Conclusion

The otologist should rule out any suspicion of multiple localizations of abnormal mobile windows in the same ear, especially when the clinical presentations appear “atypical” before performing plugging-type surgery. Awareness of the existence of new OCD variants must be systematically raised among all ENT specialists, especially radiologists, but also among audiology professionals. They also need to be aware of possible multiple localizations of OCDs in cases of “atypical” clinical presentations. Future studies and modeling should allow the development of therapeutic strategies to be adopted in cases of multiple OCDs. The acoustic energy shunt known in the classic pathomechanism model of TMWA applied in SSCD could be different and, therefore, not applicable in the case of multiple OCDs. In this case, the dispersion or depredation of acoustic energy is more likely to be diffuse.

## Author contributions

EI and PR have equal contributions in writing the manuscript. EI literature research and multiple OCDs localizations concept. GG and HT-V supervison and analysis. AL-B and EI imaging review. MD and RH analysis and interpretation. GG and RH critical review. All authors contributed to the article and approved the submitted version.
