# Supplementary material for: Why should multiple dehiscences of the otic capsule be considered before surgically treating patients with superior semicircular canal dehiscence? A radiological monocentric review and a case series
Source: Front Neurol. 2023 Aug 8;14:1209567. doi: 10.3389/fneur.2023.1209567 (PMC10442812; doi:10.3389/fneur.2023.1209567)
Supplement: Supplementary file 1 [file Data_Sheet_1.docx]

**Additional Material I**

**A**

**
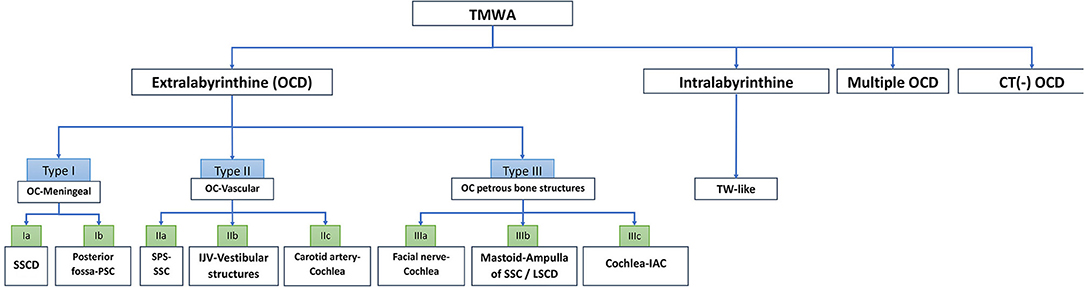
**

**B**

**
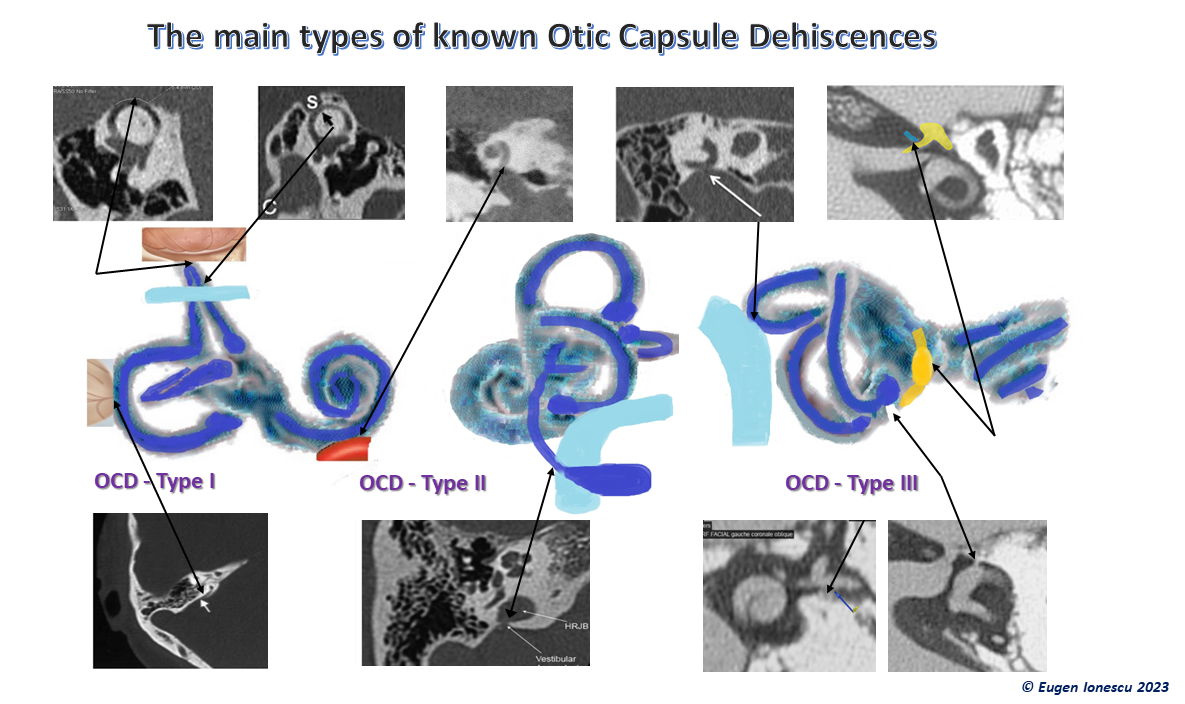
**

**Classification of Third Mobile Window Abnormalities (TMWA) (A)**

- *Extralabyrinthine ( Otic Capsule Dehiscence)* ***(B)****:*

**Type I** *(interface: membranous labyrinth-meningeal)* with two main subsets : a) The Superior Semicircular Canal in contact with the dura of the middle cerebral fossa and, b) The Posterior Semicircular Canal in contact or very close to the dura of the posterior cerebral fossa;

**Type II** *(interface: membranous labyrinth - vascular)* - contact between the inner ear membranous structures and a vascular venous or, less frequently, arterial structure;

***Type III*** *(interface: membranous labyrinth - peri petrosal structures)* : the most common variants involving a contact between inner ear membranous structures and the facial nerve canal first or second segments (e.g. cochlear-facial dehiscence (CFD I and II). Less common variants involve a contact between membrane elements of the inner ear and cells or peri petrosal bone structures such as hyperpneumatized mastoid air cells communicating with the tympanic cavity or dehiscence over the internal auditory canal.

- *Intralabyrinthine TMWA* refers to variants in which may coexist developmental or structural acquired defect of inner ear structures (as for example a dilated endolymphatic sac in contact with the vestibular aqueduct, a perilymphatic fistula, a congenital dehiscence of the stapes footplate or intralabyrinthine schwannomas)
- *Multiple OCD:* refers to associations between Type I and Type II or Type III sub variants localizations on the same ear and/or patient;
- *Not Identified OCD (or CT-TMWA)*: applies to patients who present with obvious TMWS and audio-vestibular signs, but in whom HRCT of the petrosal bone remains normal; all variants known today were obviously CT - before being reported.

**Additional material II**

Example of bilateral triple OCD localization in a patient presenting with bilateral sensorineural hearing loss, pulsatile tinnitus and very low efficacy of his hearing aids

**
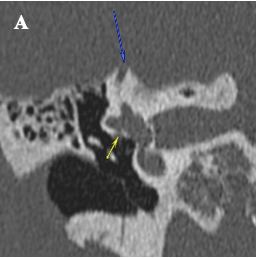

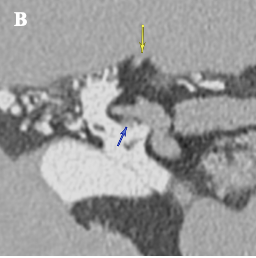

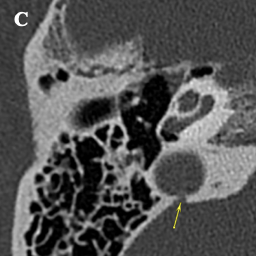
**

**
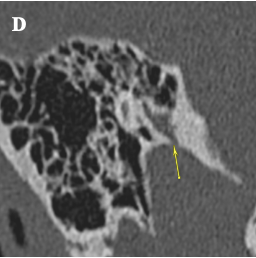

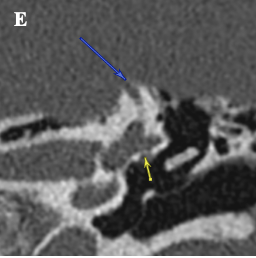

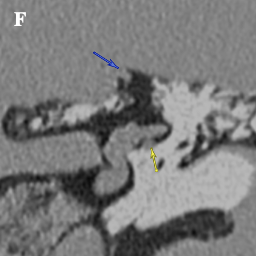
**

**
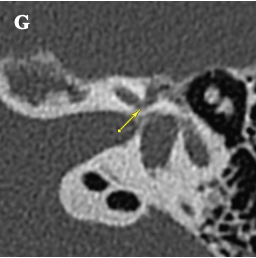

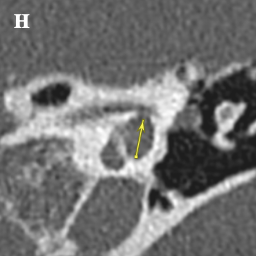
**

On the right ear in the coronal plane **(A)** there are a SSCD (*blue arrow*) (Type I) associated to a Type III CFD II (between the LSC and the NF second intra-petrosal segment) (*yellow arrow*) - same image in gray inverted contrast for a better viewing **(B)** ; axial **(C)** and coronal **(D)** view *(yellow arrows)* of a third OCD localized on the right ear (Type II or labyrinthine/Vascular type between the Vestibular Aqueduct and the right Internal Jugular Vein); On the left ear there are also three OCDs localizations on the same side: SSCD (Type I) *(blue arrow)* and CFD II (Type III) *(yellow arrow)* visible on the coronal incidence **(E )** normal or with gray inversion contrast **(F)**, while a third OCD ( CFD I - Type III) is well visible in both axial **(G)** and coronal incidences **(H)** *(yellow arrow)*.

**Additional Material III**

| **Case** | **Age (years)** | **Sex** | **Preoperative Clinical findings** | **OCD type** | **cVEMP threshold (dB HL) before surgery** | **Hearing Loss** | **Surgery/ approach**  **Material used for surgery** | **Postoperative clinical and audiological findings** |
| --- | --- | --- | --- | --- | --- | --- | --- | --- |
| 1 | 75 | M | LE:  -Hyperacusis  -Tullio P  -Pulsatile tinnitus | LE:  SSCD I  +  CFD II | RE: 95  LE: 80 | Bilateral SNHL (LE>RE) | Transmastoid  - LE SSCD occlusion  - Fascia + bone dust + fibrin glue | -Recurrence of TP and pulsatile tinnitus  -cVEMP theshold normalised |
| 2 | 74 | M | RE:  -Autophony  -Tullio P | RE:  SSCD  +  VA/IJV  +  LSC/FN | RE: 50  LE: 95 | bilateral SNHL (LE<RE)  No CHL | Middle fossa  - RE SSCD occlusion  - Fascia  temporalis + bone wax +  bone dust | -PTA threshold degradation  -Autophony reappearance  -cVEMP not controlled |
| 3 | 70 | M | RE:  -Autophony  -Pulsatile tinnitus  -Persistent dizziness  - Tullio P | RE:  SSCD  +  CFD I | RE: 70  LE: 95 | RE: CHL  Bilateral presbycusis | Transmastoid approach  - RE SSCD occlusion  - Fascia temporalis  + bone dust + fibrin glue | -Increased resonance and autophony  - Increased mixted hearing loss  - cVEMP dissapeared |
| 4 | 32 | F | LE (symptomatic):  -Pulsatile tinnitus  -Autophony  - Internal noise perception  -Positional vertigo with negative Dix -Hallpike manoever | RE:  Minimal SSCD  +  VA/IJV  +  CFDII  LE:  Large SSCD  +  CFDII | RE: 80  LE: 60 | LE: CHL | Transmastoid  -LE SSCD occlusion plugging  - Fascia temporalis +  bone dust + fibrin blue | -Increased pulsatile tinnitus and dizziness  - Internal noise perception accentuated on the operated ear  -PTA degradation bilaterally  -cVEMP normalized on the operated side |
| 5 | 58 | M | LE:  -Autophony  - Internal noise perception  -Pulsatile tinnitus | LE: -SSCD  RE:  -SSCD  -VA/IVJ | RE: 70  LE: 60 | LE: CHL | Middle fossa  - LE occlusion  - Bone wax, bone dust, fibrin glue | -New tinnitus on the left ear  - No other symptoms  - cVEMP nomalized on the operated ear |
| 6 | 49 | M | LE:  -Autophony  -Bilateral pulsatile tinnitus LE>RE  - Exercise induced dizziness | LE:  -SSCD  -CFD I  RE: -near SCD | RE: 95  LE: 50 | Bilateral CHL (LE>RE) | No Surgery | **X** |
